# Metagenomic Analysis Revealing the Impact of Water Contents on the Composition of Soil Microbial Communities and the Distribution of Major Ecological Functional Genes in Poyang Lake Wetland Soil

**DOI:** 10.3390/microorganisms12122569

**Published:** 2024-12-13

**Authors:** Yuxin Long, Xiaomei Zhang, Xuan Peng, Huilin Yang, Haiyan Ni, Long Zou, Zhong’er Long

**Affiliations:** Nanchang Key Laboratory of Microbial Resources Exploitation & Utilization from Poyang Lake Wetland, College of Life Sciences, Jiangxi Normal University, Nanchang 330022, China; longyuxin04@163.com (Y.L.); yanghl@jxnu.edu.cn (H.Y.); nihaiyan16@163.com (H.N.);

**Keywords:** Poyang Lake wetland, soil moisture, metagenomics, microbial community, functional gene

## Abstract

Poyang Lake is the largest freshwater lake in China, which boasts unique hydrological conditions and rich biodiversity. In this study, metagenomics technology was used to sequence the microbial genome of soil samples S1 (sedimentary), S2 (semi-submerged), and S3 (arid) with different water content from the Poyang Lake wetland; the results indicate that the three samples have different physicochemical characteristics and their microbial community structure and functional gene distribution are also different, resulting in separate ecological functions. The abundance of typical ANME archaea *Candidatus Menthanoperedens* and the high abundance of *mcrA* in S1 mutually demonstrate prominent roles in the methane anaerobic oxidation pathway during the methane cycle. In S2, the advantageous bacterial genus *Nitrospira* with ammonia oxidation function is validated by a large number of nitrification functional genes (*amoA, hao, nxrA*), manifesting in that it plays a monumental role in nitrification in the nitrogen cycle. In S3, the dominant bacterial genus *Nocardioides* confirms a multitude of antibiotic resistance genes, indicating their crucial role in resistance and their emphatic research value for microbial resistance issues. The results above have preliminarily proved the role of soil microbial communities as indicators predicting wetland ecological functions, which will help to better develop plans for restoring ecological balance and addressing climate change.

## 1. Introduction

Soil microorganisms are momentous components of both natural and managed ecosystems [1]. Each gram of soil may contain thousands of microorganisms, including bacteria and some small prokaryotes, fungi, and viruses. Soil microbes, especially those found in wetlands, drive the cycling and transformation of soil organic carbon (SOC) and other nutrients, promote the flow of chemical energy and information [2], and also participate in processes such as pollutant degradation and environmental remediation [3], playing a significant role in maintaining the balance and stability of wetland ecosystems [4]. Changes in wetland environmental conditions, such as nutrient content, water content, pH, vegetation, etc., can affect the structure and function of wetland microorganisms [5,6].

Metagenomic next-generation sequencing (mNGS) refers to a metagenomic research method that uses next-generation sequencing technology to directly study the composition of microorganisms in samples through high-throughput sequencing and data analysis techniques [7]. mNGS breaks through the limitations of traditional microbiology based on culture and identification and can obtain almost all DNA or RNA sequences in the sample. Based on conventional research methods such as specific target molecule amplification, restriction enzyme digestion, and electrophoresis, researchers have expanded the Sanger sequencing method and established a metagenomic research method for 16S rRNA clone library sequencing [8]. The emergence of mNGS has made metagenomic methods a popular tool in microbial research. Its advantages include independent cultivation, relatively simple operation, and theoretically unbiased detection of all microorganisms in the sample. Although there are problems such as the large amount of data obtained, complex analysis methods and processes, and difficulty in unifying and standardizing experimental and data analysis processes [9,10], the genetic material of microbial communities can be identified through subsequent optimization analysis methods, which can fully explore their genetic diversity and biochemical reactions, and even further combine with environmental variables to explore the response mechanism between microorganisms and the environment.

Wetlands are one of the most productive and valuable ecosystems in the world, providing 40% of the global ecosystem service value [11,12,13,14]. Poyang Lake is located in the middle and lower reaches of the Yangtze River and is the most typical large-scale shallow-water lake in China [15]. The habitat types and structures of the intertidal wetlands are diverse, providing a vast and diverse habitat for organisms. Poyang Lake has also become an important biological resource reservoir in China’s subtropical regions [16,17]. The unique hydrological rhythm of alternating flood and dry seasons causes periodic inundation and exposure of intertidal habitats, which is an important driving force affecting the species composition and microbial diversity of plant communities in floodplain lakes and intertidal wetlands [18]. In recent years, against the backdrop of increased human activity and interference, the water level of Poyang Lake has become low and withered [19,20,21,22], the duration of flooding has become shorter, and the beaches have been exposed earlier, leading to changes in the microbial community in the soil [23]. Exploring the changes in biodiversity and functional gene abundance of communities can effectively evaluate the ecological effects caused by changes in hydrological conditions, which is of great significance for maintaining and protecting biodiversity in Poyang Lake and predicting the distribution pattern evolution of microbial communities under future water level fluctuations. As of now, it is unclear how changes in soil moisture content in Poyang Lake wetland affect the functionality of microbial communities. Therefore, it is necessary to conduct in-depth analysis of the main functions driven by microorganisms in different soil moisture contents.

It is generally reasonable to believe that (1) there are significant differences in microbial community structure and physicochemical factors among soil samples with different moisture contents; (2) there are differences in the distribution characteristics of carbon, nitrogen, and antibiotic resistance genes in soil samples with different moisture contents; (3) there is a profound relationship between microbial communities and the driving processes of underground ecological functions. However, the impact of soil moisture on soil microbial community structure and the distribution of important ecological genes varies in different wetland ecosystems. Therefore, the microbial composition and abundance of three soil samples with different water contents from Poyang Lake wetland were analyzed using metagenomic technology, and their effects on the distribution of important ecological functional genes were investigated to reveal the interrelationships between soil water content, soil microbial community structure, and important ecological functional genes.

## 2. Materials and Methods

### 2.1. Soil Sampling

**Figure 1:**
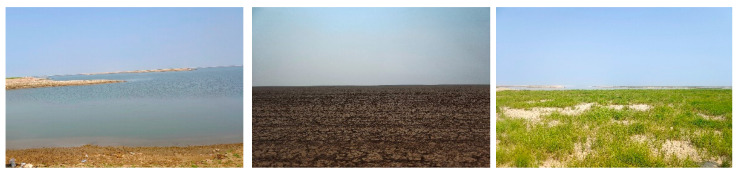
The soil sampling sites of different water contents in Poyang Lake wetland successively showing S1, S2, S3 from left to right.

In October 2022, soil samples were collected from the Poyang Lake region in the range of E 116°25′ and N 28°58′. Three different sampling points (S1 of E 116°25′20.45″ and N 28°58′32.91″, S2 of E 116°25′21.72″ and N 28°58′35.49″, S3 of E 116°23′43.94″ and N 28°58′15.74″) of the Poyang Lake wetland were selected for soil sampling, S1 was submerged by lake water (sedimentary state) and had the highest water content, S2 had a high water content (semi-submerged state), S3 had the lowest water content and was covered with plants (drought), and the sample soil reality is shown in Figure 1. Soil samples were taken at a depth of 10 cm from the surface with an aseptic spatula, plant material and detritus were removed during sampling, and three biological replicates were performed for each sampling point. The sample is named according to its source, for example, the sample from the S1 sampling point is named the S1, and 3 samples taken from sampling point S1 were denoted as S1-1, S1-2, and S1-3, respectively, and so on. A total of 9 samples were collected. Each sample was separated into two groups: one was dried at room temperature for soil physicochemical property measurement and the other was stored at −20 °C for DNA extraction.

### 2.2. Measurement of Soil Physicochemical Properties

The water content (WC) of the soil samples was identified by dividing the mass difference between fresh and dry soil of each sample by the mass of the dry soil, which was obtained by heating the soil sample at 105 °C for 24 h. Soil pH was measured using a pH meter (Leici PHSJ-4F, Shanghai, China) in a 1:5 soil/water suspension after shaking at 25 °C for 1 h in an incubator shaker at 5000 r/min. Soil total carbon (TC) was determined using an element analyzer (Sercon Integra 2, Crewe, UK), and organic carbon (SOC) was determined by the potassium dichromate volume-external heating method. Total nitrogen (TN) was determined by an automatic nitrogen analyzer (SKD-1100, Shanghai, China). Ammonium nitrogen and nitrate nitrogen were extracted by potassium chloride solution-spectrophotometry. Nitrate nitrogen was calculated by ultraviolet spectrophotometry. Three soil replicates from each sampling site were used for physicochemical analysis, and the average and standard deviation of three replicate determinations were calculated.

### 2.3. DNA Extraction, Sequencing, and Data Processing

The macrogenomic DNA was extracted from 9 soil samples, respectively, using the DNeasy powersoil kit (QIAGEN, Hilden, Germany) following the manufacturer’s protocol, and the quality and concentration of DNA were monitored by Nanodrop spectrophotometer, Qbuit fluorometer, and agarose gel electrophoresis.

Prepared DNA samples were sent to BENAGEN (Wuhan, China) for shotgun metagenomics sequencing. A total of 9 samples were sequenced, respectively, by second-generation Illumina to obtain raw reads. Fastp software (https://github.com/OpenGene/fastp, accessed on 10 May 2023, Version 0.23.2) was employed to remove low-quality tags and to obtain high-quality sequencing data (Clean Tags). MEGAHIT software (https://github.com/voutcn/megahit, accessed on 14 May 2023, Version 1.2.9) was used for metagenomic assembly, and contig sequences shorter than 300 bp were filtered. We used MetaGeneMark (http://exon.gatech.edu/meta_gmhmmp.cgi, accessed on 27 May 2023, Version 3.26) software’s default parameters to identify the coding regions of the genome, acquired with the results of single sample assembly gene prediction, and obtain the translated protein sequence. We used MMseqs2 (https://github.com/soedinglab/mmseqs2, accessed on 10 June 2023, Version 12-113 e3) software to remove redundancy, with a similarity threshold set to 95% and a coverage threshold set to 90%, to build the redundant data sets.

### 2.4. Bioinformatics Analysis

Non-redundant gene sets analysis was performed on the BMKCloud (http://www.biocloud.net/, accessed on 4 April 2024), which is a biological information cloud platform. The abundance of biotaxa and species in each sample was obtained by comparing the protein sequences of non-redundant genes with the Nr database using BLAST (Version 2.14.0). The functional annotation information was obtained by comparing the protein sequences of non-redundant genes with KEGG, eggNOG, GO, and CAZy carb enzyme databases using BLAST.

### 2.5. Statistical Analysis

The above results revealed the different samples’ overall functional profiles. Using functional gene information from the relevant literature, we extracted all annotated genes and drew a histogram of gene abundance for the carbon and nitrogen cycling processes in different pathways using ORIGIN mapping software (Version Origin 2021). Analysis of significant differences between and inter-groups was performed with SPSS software (Version IBM SPSS Statistics 27), tested by one-way analysis of variance (ANOVA). *p* < 0.05 was considered to indicate a statistically significant difference. The language R (Version 4.3.0) was used to create a heatmap showing the correlation between microbial functional genes involved in carbon and nitrogen cycling and soil physicochemical factors.

## 3. Results

### 3.1. Physicochemical Properties of Soil Samples

The soil metagenomic sequencing in the Poyang Lake wetland exhibited good quality, and its basic information is displayed in (Supporting Information Table S1). The physicochemical properties measured in the study showed that the three samples reflected significantly different soil structures (Table 1). The water content is the major factor studied in this article, and it gradually decreases from sample S1 to sample S3. Among the various chemical indicators measured, the contents of NH_4_^+^-N represented the highest abundance in S1, and then, in order, S3 and S2, but there is no significant difference between them. All other indicators, such as NO_3_-N, TC, TN, and SOC, exhibited significant differences between different samples and displayed similar trends of change, including that, as the moisture content decreases, each indicator first increases and then decreases, all showing the highest abundance in S2, and there are significant differences among the three samples. However, the soil C/N of S1 is similar to that of S3 and there is no difference between them, with S2 being the highest sample.

The correlation heat map between physical and chemical properties (Supporting Information Figure S1) indicates that the soil moisture content is negatively correlated with ammonium nitrogen, nitrite, and pH, and positively correlated with other physicochemical factors.

### 3.2. Taxonomic Composition of Microbial Communities

The analysis results of the soil microbial diversity index at each sampling point in the Poyang Lake wetland are shown in Table 2. The Observed Species, ACE, and Chao1 indices, which represent the species richness in soil, revealed that the microbial diversity in the three samples showed the same trend, with S2 having the highest species richness and S1 having the lowest. The Pielou index was used in the Alpha index to measure the evenness of species diversity distribution, with species distribution in S1 being the most uniform. Shannon and Simpson reflect the diversity of a community, which is influenced by the richness and evenness of species in the sample community. The larger the Shannon index, the higher the diversity of the sample community, with S1 having the highest value.

The composition and abundance of soil microorganisms in different samples are shown in Supporting Information Figure S2, which indicates that a total of 4 domains, 7 kingdoms, 199 phyla, 832 families, 3470 genera, and 23813 species were identified by annotating the total microbial species of three collected soil samples. The prokaryotic microbial communities are mainly composed of bacteria (76.05–89.88%), with a relatively small proportion of archaea (1.32–9.60%). The eukaryotic microbial community is mainly composed of fungi (0.01–0.14%). In addition, the annotation results of the metagenome contain a large number of high-quality sequences (7.23–20.00%) that have not been classified, indicating that there is still a large amount of unknown biological information for exploration.

**Figure 2:**
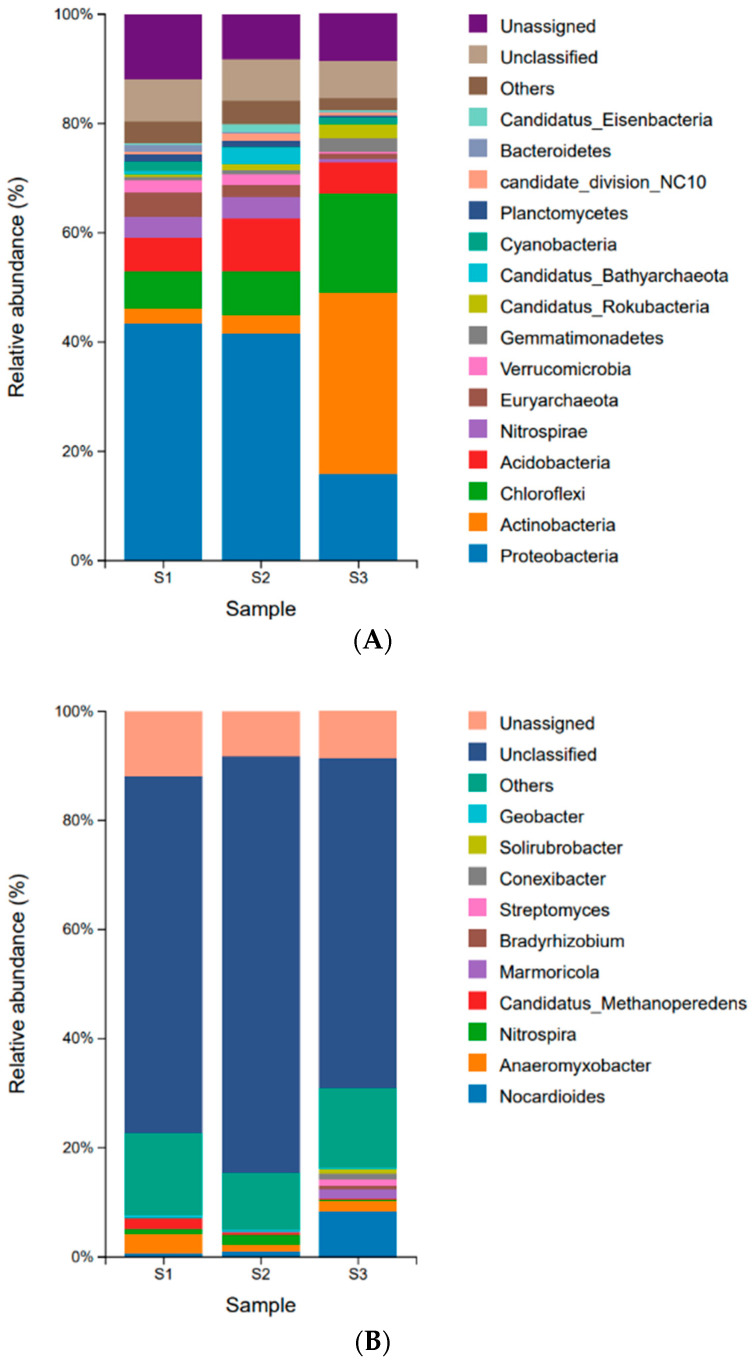
Microbial community composition. (**A**) Relative abundance of the major phyla based on metagenomic sequences in the Poyang Lake wetland, (**B**) Relative abundance of the major genus. Unassigned: A sequence that has not been accurately identified or classified as a known biological species. Unclassified: Although DNA sequences or genes have been identified to a certain level, they cannot be clearly localized to specific biological taxonomic units at more specific taxonomic levels. Other: Species with relatively low abundance.

Microbial communities vary significantly among soil samples with different water contents, displaying similar classifications but varying abundances among species. At the phylum level (Figure 2A), bacteria are divided into 199 phyla, mainly including Proteobacteria, Actinobacteria, Chloroflexi, Acidobacteria, Nitrospirae, Verrucomicrobia, Gemmatimonadetes, Candidatus Rokubacteria, Cyanobacteria, etc. Among them, Proteobacteria is the dominant phylum in the S1 and S2 samples, accounting for 43.49% and 41.65%, respectively. Actinobacteria is the dominant phylum in S3, accounting for 33.24%, which is 12.8 and 10.4 times higher than its abundance in S1 and S2, respectively.

At the genus level (Figure 2B), there are a total of 3470 genera in the annotated classification of bacteria, mainly including *Nocardioides*, *Anaeromyxobacter*, *Nitrospira*, *Candidatus Methanoperedens*, *Marmoricola*, *Bradyrhizobium*, etc. The dominant genus in S1 is *Anaeromyxobacter* (3.46%), while it is *Nitrospira* in S2 (1.85%) and Nocardia-like (8.25%) in S3. The relative abundance of *Candidatus Methanoperedens* with the second abundance of S1 is 1.77%, which is 7.1 and 8.4 times higher than that of S2 and S3, respectively. The microbial diversity of archaea communities is significantly lower than that of bacterial communities. It was found that the most abundant archaea are *Euryarchaeota*, which account for 2.58% of the total microbial taxonomic level, and *Bathyrachaeota* account for 1.26% of the total microbial taxonomic level.

**Figure 3:**
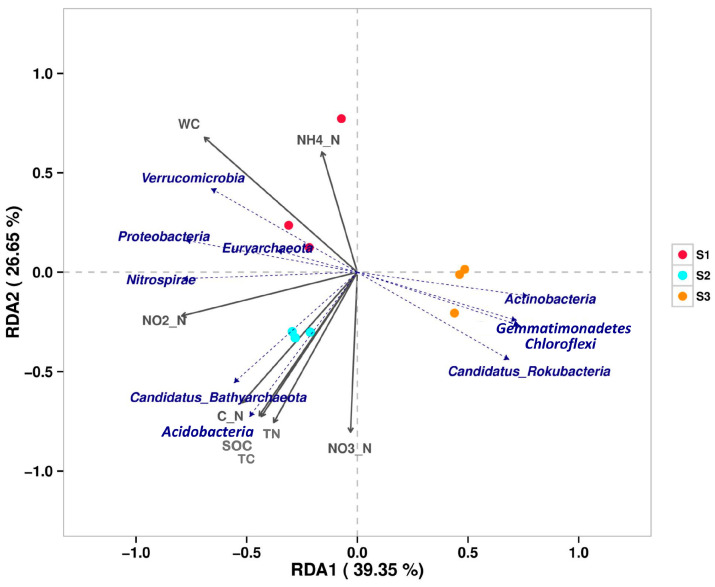
RDA analysis is used to reflect the correlation between physicochemical factors and microbial species (phylum level) in the soil of the Poyang Lake wetland. The dashed arrow in the figure represents the level of microbial phylum, while the solid arrow represents physical and chemical factors. The arrow representing microbial species is closer to a certain physicochemical factor, indicating that the physicochemical factor has the greatest impact on that species’ abundance. C_N represents the carbon-to-nitrogen ratio of the soil.

In the correlation analysis between microbial phylum level and physicochemical factors, the cumulative interpretation rates of axis one and axis two in the RDA graph are 66% (Figure 3). Three samples are clustered separately and there are significant differences among them. The soil moisture content (WC) is positively correlated with Verrucomicrobia, Proteobacteria, and Euryarchaeota, and negatively correlated with Actinobacteria, Gemmatimonadetes, Chloroflexi, and Candidatus Rokubacteria. Nitrospirae is positively correlated with nitrite in soil. The content of soil C/N, organic carbon, total carbon, and total nitrogen is positively correlated with Candidatus Bathyachiaeota and Acidobacteria.

### 3.3. Microbial Carbon Fixation Genes

The assimilation of CO_2_ into organic material is quantitatively the most important biosynthetic process on Earth [24,25,26]. Six autotrophic CO_2_ fixation pathways have been found in various environments to date: including aerobic pathways, such as the Calvin cycle (CBB), 3-hydroxy propionic acid dual cycle (3-HP), and 3-hydroxy propionic acid cycle/4-hydroxybutyric acid cycle (3HP/4HB), and anaerobic pathways, such as the reducing tricarboxylic acid cycle (rTCA), reducing acetyl CoA pathway (WL), and dicarboxylic acid/4-hydroxybutyric acid cycle (DC/HB). The enzymes catalyzing limiting steps in a given pathway are usually conserved and act as key enzymes, and the corresponding coding genes, often named marker genes [27], are commonly used in microbial ecological studies. In this study, *cbbL* was used for ribulose 1,5-bisphosphate carboxylase/oxygenase (RubisCO) of the CBB pathway, *aclA* for the ATP citrate lyase in the rTCA pathway, *acsA* for the carbon-monoxide dehydrogenase catalytic subunit in the WL pathway, *accA* for the acetyl-CoA carboxylase carboxyl transferase subunit alpha in the 3HP/4HB pathway, *pcc* for the Malonyl-CoA Reductase in the 3-HP pathway, and *hcd* for the 4-hydroxybutyryl-CoA dehydratase in the DC/HB pathway.

**Figure 4:**
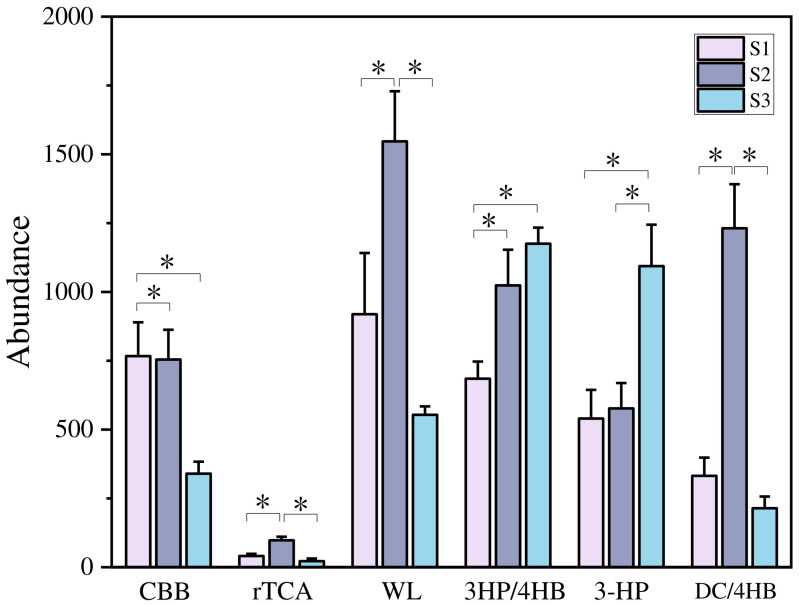
The abundance of key enzymes involved in six carbon fixation pathways in three samples. * Marks the significance of differences (*p* < 0.05).

Six marker genes for carbon fixation pathways were detected in three samples with different soil moisture contents (Figure 4). Among them, the abundance of the marker gene *aclA* in the rTCA pathway was the lowest in all three samples. The *acsA* in the WL pathway had the highest abundance in samples S1 and S2, while the *accA* gene in the 3HP/4HB pathway had the highest abundance in sample S3.

### 3.4. Microbial Methane Cycling Genes

Methane metabolism, driven by methanogenic and methanotrophic microorganisms, plays a pivotal role in the carbon cycle [14,28,29]. The key enzyme-corresponding coding genes in the natural methane cycle pathway driven by microorganisms are as follows [30,31]: *mcr*, methyl coenzyme-M reductase gene; *mtr*, tetrahydromethanopterin S-methyltransferase gene; *mer*, F_420_-dependent methylenetetrahydromethanopterin dehydrogenase gene; *mtd*, methylenetetrahydromethanopterin reductase gene; *mch*, methenyltetrahydromethanopterin cyclohydrolase gene; *ftr*, formylmethanofuran-tetrahydromethanopterin N-formyltransferase gene; *fwd*, formylmethanofuran dehydrogenase gene; *fdo*, formate dehydrogenase iron-sulfur subunit gene; *fdh*, formate dehydrogenase gene; *mdh*, malate dehydrogenase gene; *pmo*, methane/ammonia monooxygenase.

**Figure 5:**
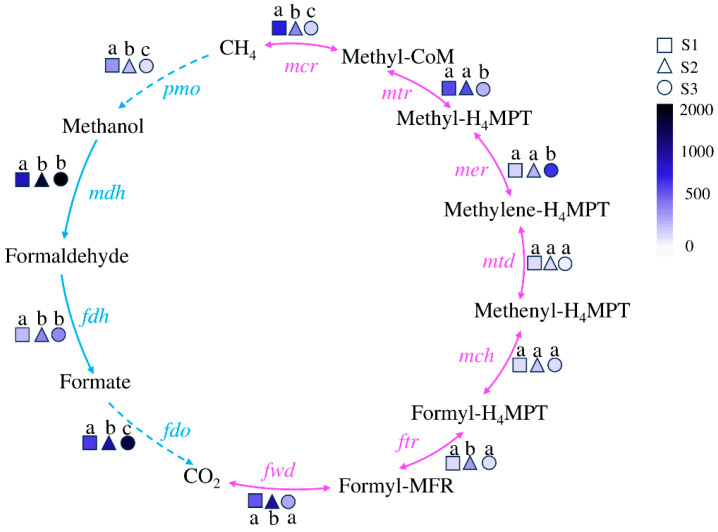
The abundance of methane cycling and functional genes driven by soil microorganisms in Poyang Lake wetland. The pink line represents the process of methane generation, and the blue line represents the process of methane oxidation. The genes on the arrows are the functional genes of the key enzymes in this turnover process. The different shapes represent different samples, and the filling colors from light to deep represent the abundance of the gene from low to high, while the letters “a”, “b”, and “c” indicate significant differences between samples.

The distribution of methane metabolism functional genes in soils with different water contents is shown in Figure 5. The KEGG gene functional annotation indicates that the functional genes of each step in the methane cycle are distributed in all three samples, forming a complete cycle. The genes related to methane metabolism in S1 are concentrated in the anaerobic process, and their abundance is higher than that in S3, except for the *mer* gene. The genes related to methane metabolism in S3 are concentrated in the aerobic pathway, and their abundance is higher than that in S1, (except for the *pmo* gene). S2, whether in aerobic or anaerobic conditions, has a relatively average distribution of functional genes.

### 3.5. Microbial N Cycling Genes

Microbial nitrogen cycling mainly involves six pathways [32,33,34], each of which has corresponding key enzymes; the marker genes coding by microorganisms for each key enzyme are as follows [35,36]: nitrogenase gene *nifH* in nitrogen fixation; ammonia monooxygenase gene *amoA/amoB*, hydroxylamine oxidoreductase gene *hao,* and nitrite oxidoreductase gene *nxrA/nor* in nitrification; nitrate reductase gene *narG*, nitrite reductase gene *nirK/nirS*, nitric oxide reductase gene *norB,* and nitrous oxide reductase gene *nosZ* in denitrification; N_2_H_4_ synthase gene *hzsA* and N_2_H_4_ oxidoreductase *hzo* in anaerobic ammonia oxidation; nitrate assimilation reductase gene *nasA/narB* and nitrite assimilation reductase gene *nirA/nirB* in assimilation nitrogen reduction; nitrate dissimilatory reductase gene *napA*, and nitrite dissimilatory reductase gene *nrfa* in dissimilar nitrogen reduction.

**Figure 6:**
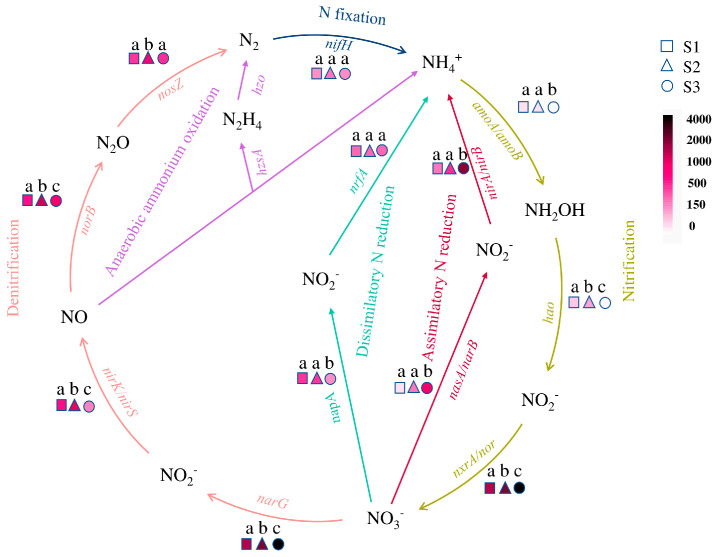
The nitrogen cycling driven by soil microorganisms and the abundance of its functional genes in the Poyang Lake wetland. Different colored arrows represent different nitrogen cycling processes in soil, and the genes on the arrows are the functional genes of the key enzymes in this turnover process. The different shapes represent different samples, and the filling colors from light to deep represent the abundance of the gene from low to high, while the letters “a”, “b”, and “c” indicate significant differences between samples.

The presence of key enzyme genes related to the nitrogen cycling pathway in three soil samples is shown in Figure 6. Except for the key enzyme genes in anaerobic ammonia oxidation that have not been discovered yet, other pathways exist in all three samples. It can be seen that the nitrogen cycling profiles of three different soil states varied with changes in soil moisture content, the abundance of key enzyme genes related to anaerobic ammonia oxidation in S3 is extremely low and can be ignored. The functional genes driving nitrogen cycling by microorganisms in S1 and S2 are significantly enriched in the Denitrification and Dissimilatory N reduction processes, while S3 is enriched in the Nitrification and Assimilatory N reduction processes.

### 3.6. Microbial Antibiotic Resistance Genes

Antibiotic resistance genes are a new type of environmental pollutant widely present in environmental microorganisms and media [37]. Antibiotic resistance genes in the environment can not only replicate and increase with the proliferation of microorganisms but also migrate and spread between different microorganisms, directly or indirectly affecting ecological security and human health [38,39].

**Figure 7:**
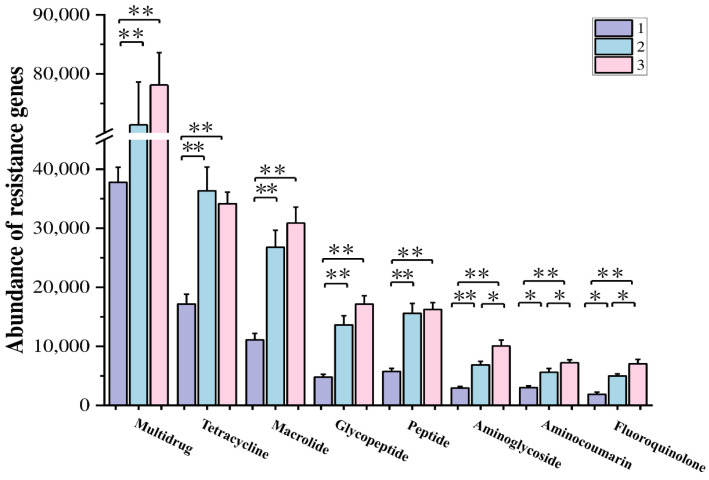
The abundance of resistance genes secreted by microorganisms in soils with different moisture contents in Poyang Lake wetland. “*” means significant differences (*p* < 0.05) and “**” means extremely significant differences (*p* < 0.01).

We analyzed the top eight antibiotic-resistance genes in the abundance of soil microorganisms in Poyang Lake wetland (Figure 7). The most abundant resistance gene type among the three samples is multidrug. Except for tetracycline-resistance genes, which have the highest abundance in S2, the abundance of other antibiotic-resistance genes is highest in S3.

### 3.7. The Correlation Between C/N-Cycle Functional Genes and Physicochemical Factors

**Figure 8:**
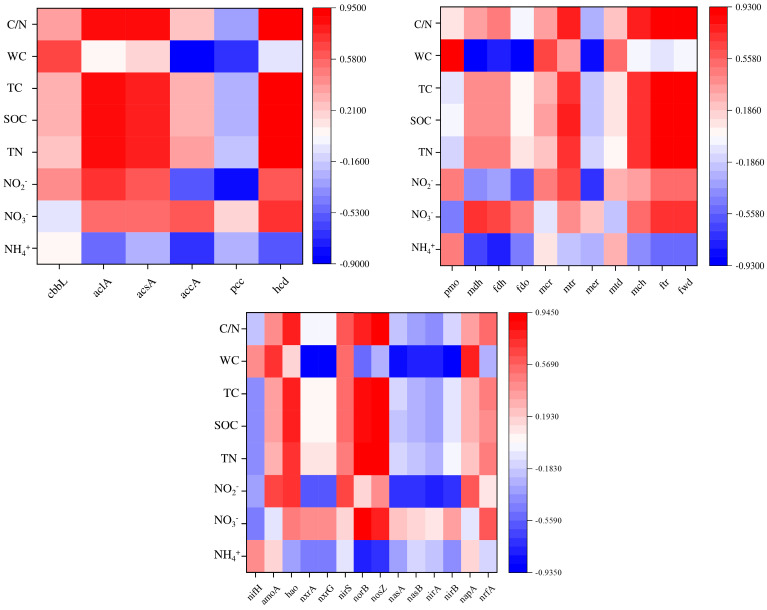
Correlation heatmap between soil physicochemical factors and genes related to carbon decomposition, methane cycle, and nitrogen cycle. Red represents a positive correlation, blue represents a negative correlation. The darker the color, the stronger the correlation.

Correlation analysis was conducted between carbon, nitrogen, and methane cycling, relating functional genes driven by microorganisms in three samples with different moisture contents of the soil and soil physicochemical properties (Figure 8). The abundance of soil functional genes in different states was significantly or extremely significantly correlated with environmental factors. Among them, water content is a key factor affecting soil carbon and nitrogen ecological functions driven by microorganisms.

In the carbon fixation pathway, the abundance of *cbbl* is significantly positively correlated with water content, while the abundance of *aclA*, *acsA*, and *hcd* is highly significantly positively correlated with C/N, total carbon, total nitrogen, and organic carbon content, and negatively correlated with ammonia nitrogen content. The abundance of *accA* and *pcc* is significantly negatively correlated with water content and nitrite nitrogen content, and *accA* is also significantly negatively correlated with ammonium nitrogen content.

During the methane cycle, there is a highly significant positive correlation between *pmo* abundance and water content. In anaerobic processes, the *mdh*, *fdh*, and *fdo* are significantly negatively correlated with water content, negatively correlated with nitrite and ammonium nitrogen, and positively correlated with nitrate. During aerobic processes, *mcr* and *mtd* are positively correlated with water content. *mtr*, *mch*, *ftr*, and *fwd* are significantly positively correlated with C/N, total carbon, total nitrogen, and organic carbon content, positively correlated with nitrite and nitrate content, and negatively correlated with ammonium nitrogen content.

During the nitrogen cycling, there are a significant positive correlation between *amoA* during ammonia oxidation and water content and nitrite, while *hao* is significantly positively correlated with C/N, total carbon, total nitrogen, organic carbon, and nitrite content; During denitrification, *norB* and *nasZ* are significantly positively correlated with C/N, total carbon, total nitrogen, organic carbon, and nitrate content, and significantly negatively correlated with ammonium nitrogen; The assimilation and nitrogen reduction processes of *nasA*, *narB*, *nirA*, and *nirB* are significantly negatively correlated with water content and nitrite.

## 4. Discussion

Wetlands, as an important factor in ecosystems, play a critical role in regulating climate change as a carbon sink and a carbon source, provide a unique habitat, and support biodiversity [40]. The Poyang Lake is the largest freshwater lake in China, and one of the largest freshwater lake/wetland complexes in Asia, which plays a momentous role in regional and global biodiversity conservation [41,42]. In this study, high-throughput sequencing was performed to analyze the microbial characteristics in 3 soil samples with different water content and to dissect the interactions between the microbial community’s functional structure, ecological responses, and biogeochemical processes.

### 4.1. The Difference in Moisture Content Results in Differences in the Physicochemical Properties of Wetland Soil

Wetlands serve as a medium for receiving water and drainage, and hydrological conditions are the fundamental attributes of wetland ecosystems [43]. Water conditions determine the physical properties of wetland soil, including the type and the structure of surface plant communities and the soil microbial community, affecting ecosystem productivity [44].

The physicochemical properties of 3 soil samples with different moisture from the Poyang Lake wetland were determined in this study (Table 1). It was found that the soil from all three sampling points was weakly acidic; therefore, pH value is not a key influencing factor on the physicochemical properties of the Poyang Lake wetland. Among, except for ammonium nitrogen, all other forms of carbon and nitrogen content, soil C/N ratio are highest in S2. The higher C/N ratio in S2 means better soil quality under this moisture content condition, indicating that excessive or insufficient soil moisture in wetlands is not conducive to the accumulation of their carbon and nitrogen content.

Similarly to our research findings, previous studies have also demonstrated that water conditions are a key factor affecting wetland soil properties. The study on soils from the East Dongting Lake wetland, China showed that the water content can control the structure and function of wetlands, and changes in water content can alter the archaeal distribution patterns [45]; The different water conditions in East Dongting Lake wetlands jointly affect the soil microbial biomass carbon, nitrogen, and enzyme activities [46].

### 4.2. The Difference in Soil Physicochemical Properties Results in Differences in Soil Microbial Community Structure

Thanks to the diverse environmental conditions, the soil contains the most diverse microbial community on earth [47,48]. The spatiotemporal specificity of soil physicochemical properties could prompt microorganisms to evolve rich strategies to cope with extreme environments. Therefore, even slight differences in soil environment can lead to changes in the composition, quantity, and function of microbial populations [49,50,51].

The β diversity PCA diagram of soil microbial communities with different water contents from the Poyang Lake wetland revealed significant differences among the three samples (Supporting Information Figure S3). From the relative abundance of soil microorganisms in the Poyang Lake wetland (Figure 2), it can be seen that at the phylum level, S1 and S2, which are dominated by Proteobacteria, have similar microbial compositions, but there are significant differences compared to S3, which is dominated by Actinobacteria. At the genus level, all three samples contain a large number of unassigned and unclassified microbial genes, as existing microbial resources and technologies are far from sufficient to support our exploration of all microbial genes in the soil. Meanwhile, there are significant differences among the three samples. S1 forms a specific community of the dominant genus of *Anaeromyxobacter* under strict anaerobic conditions, and the second most abundant genus is *Candidatus Methanoperedens*, which can generate methane. S2 forms a specific community dominated by *Nitrospira*, which has nitrification in the nitrogen cycle, oxidizing nitrite to nitrate to provide proton power. S3 grows a specific community of aerobic actinomycetes with *Nocardiodies* as the primary bacterial genus, capable of producing multiple antibiotics. The RDA analysis of the correlation between microbial communities and soil physicochemical properties (Figure 3) manifested that among the microbial community composition of the three soils in Poyang Lake, water content, TC, TN, SOC, and soil C/N ratio are the key influencing factors on the microbial community in Poyang Lake wetland.

In the early research on the structure and function of the soil microbial community in the Poyang Lake wetland, correlation analysis and RDA analysis showed that the composition of the soil bacterial community in Poyang Lake wetland was mainly influenced by soil organic matter and nutrient elements (TOC, TN), and soil moisture content was also one of the influencing factors [52,53]. This is roughly consistent with our research results. According to Liu et al.’s study, the planktonic bacteria in the Poyang Lake wetland were most abundant in the Bacteroidetes, Actinobacteria, and Proteobacteria, and their diversity was significantly affected by hydrological rhythms [54].

In the context of extreme drought and prolonged dry seasons, the microbial community in the Poyang Lake wetland is significantly influenced by element content and water conditions, gradually forming a relatively stable and specific composition structure.

### 4.3. The Differences in Soil Microbial Community Structure Result in Differences in Ecological Function Distribution

Microorganisms, as an important component of lake wetland ecosystems, drive the cycling of nutrients and the migration and transformation of pollutants such as heavy metals in lake wetlands [55,56,57]. At the same time, the community composition and function of wetland soil microorganisms are also significantly influenced by element content, water conditions, and lake wetland management methods.

The main carbon fixation pathways of soil under different flooding conditions are different. According to the results shown in Figure 4, under long-term flooding conditions like S1, the carbon fixation pathways are mainly WL and CBB pathways. Under S2, the pathways include mainly WL and DC/4HB through anaerobic pathways, while under S3 conditions, 3HP/4HB and 3-HP through aerobic pathways are mainly involved. The CBB cycle is the most common CO_2_ fixation method in organisms [58]. The key enzyme type I rubisco enzyme (CbbL) in the CBB pathway is commonly found in green-like bacterial communities (plants, cyanobacteria, green algae, alpha proteobacteria, beta proteobacteria, and gamma proteobacteria, etc.) and red-like bacterial communities (red algae, brown algae, alpha-proteobacteria, and beta-proteobacteria, etc.) [59]. The S1 and S2 carbon fixed functional genes *cbbL* have relatively high abundance, which is speculated to be due to their high relative abundance of Proteobacteria (43.49%, 41.65%). According to results shown in Figure 8, water content, TC, TN, SOC, and C/N are key factors affecting the distribution of carbon fixation functional genes.

The anaerobic degradation of methane in nature is mainly achieved through the reverse reaction process of the methane production pathway, which is usually mediated by a type of anaerobic methanotrophic (ANME) archaea [60,61]. Previous studies have found that methyl coenzyme M reductase (Mcr) is a key enzyme for methane production and activation of alkane molecules [62]. Among them, *mcrA*, which is the coding gene for one of the two subunits of Mcr, is often used to detect the abundance and population of methane-metabolizing archaea in the environment. From the methane cycle diagram (Figure 5), it can be seen that the abundance of the *mcrA* gene varies significantly in soils under different flooding conditions, with S1 having the highest abundance and S3 having the lowest abundance. The ANME group with the highest abundance is found in S1, and Figure 2B shows the typical ANME archaea *Candidatus Menthanoperedens* with high abundance in S1. In addition, in the heat map of the correlation between methane cycle functional genes and soil physicochemical factors (Figure 8), there is a positive correlation between *mcr* and water content of nitrite, indicating a certain relationship between the anaerobic methane oxidation process involved in *mcr* and nitrite content. Therefore, the soil of Poyang Lake wetland under flooded conditions plays a prominent role in the anaerobic methane oxidation process.

The nitrification process in the nitrogen cycle is catalyzed by ammonia-oxidizing bacteria and nitrifying bacteria, respectively [63]. In 2015, both ammonia oxidation and nitrite reduction were actualized by some lineages of *Nitrospira*, known as complete ammonia oxidation (Comammox), have been found [64]. The abundance of *Nitrospira*, the dominant bacterial genus in S2, is significantly higher than that in other samples. Meanwhile, S2 has a relatively high abundance of functional genes (*amoA*, *hao*, *nxrA*) during the nitrification process. However, the abundance of *nxrA* in S3 is significantly higher than that in S2, and we speculate that this is due to the high abundance of *Chloroflexi* in S3, which also has nitrite-reducing ability. In addition, studies have shown a significant negative correlation between the abundance of *Nitrospira* and the concentration of ammonia nitrogen. In this study, S2, with the lowest ammonia nitrogen concentration (Table 1), contained the highest abundance of *Nitrospira*, which also confirms this.

### 4.4. The Differences in Soil Microbial Community Structure Result in Differences in Antibiotic Resistance Distribution

Antibiotic resistance is a global health challenge, involving the transfer of bacteria and genes between humans, animals, and the environment [65,66]. On the map of antibiotic resistance gene abundance distribution, S3 has the highest abundance except for the tetracycline-resistance gene, which is reasonable as the wetland soil is closest to human activities. In addition, the most abundant genus of *Nocardioides* in S3 has strong adaptability to relatively harsh environments and can be widely distributed by regulating intracellular metabolism, synthesizing secondary metabolites, and secreting special enzymes. Linking a high abundance of resistance genes with a high abundance of the *Nocardioides* genus can confirm the special function of S3 soil microorganisms on resistance genes.

The three hypotheses mentioned in the previous introduction have been verified in this study. Through data detection and analysis, it is confirmed that the microbial structure and physicochemical factors of soil samples with different water content in Poyang Lake wetland are different. They also have completely different functional profiles of carbon, nitrogen, and antibiotic resistance genes. Through the correlation analysis of data and literature review, the deep relationship between microorganisms and ecological functions was demonstrated. The analysis of these factors will provide important insights into microbial community structure as a worthwhile indicator of ecological functional changes.

## 5. Conclusions

The microbial community composition and physicochemical properties of the three soil samples with different moisture contents in the Poyang Lake wetland exhibit distinct characteristics, and their mutual influence leads to their different ecological functions. This study analyzed the microbial community composition’s characteristics and functional gene abundance in different soil samples of wetlands, in order to gain a deeper understanding of the relationship between underground microbial communities and field ecological features. The abundance of *Candidatus menthanoperedons* and the high abundance of *mcrA* in S1 mutually confirm the prominent role of S1 in the anaerobic oxidation pathway of methane in the methane cycle process. The dominant bacterial genera *Nitrospira* in S2 is mutually confirmed with a large number of nitrification functional genes (*amoA*, *hao*, *nxrA*), indicating the prominent role of S2 in nitrification during the nitrogen cycle. The dominant bacterial genera *Nocardia* in S3 is mutually confirmed with a large number of discovered antibiotic resistance genes, indicating the important function of S3 in resistance genes and its outstanding research value for microbial resistance issues. The above study has preliminarily confirmed the indicator role of soil microbial communities in predicting wetland ecological functions, which will help us better formulate plans for restoring ecological balance and regulating climate change. Although it is unclear whether these conclusions can be extended to other wetland ecosystem types due to increased environmental heterogeneity and climate uncertainty, understanding the reactions of soil microorganisms and their potential impacts is crucial for a deeper understanding of the functions of underground wetland ecosystems.

## Figures and Tables

**Table 1:** Physicochemical characteristics of soil samples from Poyang Lake wetland.

	Sample	S1	S2	S3
Physicochemical Property				
WC (%)		25.25 ± 1.05 (c)	11.05 ± 1.35 (b)	3.75 ± 0.65 (a)
pH		6.34 ± 0.1 (ab)	6.55 ± 0.23 (b)	6.01 ± 0.27 (a)
NH_4_^+^-N (mg/kg)		5.64 ± 0.99 (a)	2.27 ± 0.69 (a)	3.42 ± 1.22 (a)
NO_3_-N (mg/kg)		1.46 ± 0.12 (a)	10.55 ± 1.52 (c)	6.01 ± 1.15 (b)
NO_2_-N (mg/kg)		0.07 ± 0.01 (ab)	0.09 ± 0.01 (b)	0.05 (a)
TN (g/kg)		0.19 ± 0.06 (a)	1.64 ± 0.02 (c)	0.49 ± 0.09 (b)
TC (g/kg)		1.73 ± 0.57 (a)	21.38 ± 0.34 (c)	4.77 ± 1.2 (b)
SOC (g/kg)		1.59 ± 0.52 (a)	20.56 ± 0.84 (c)	4.08 ± 0.98 (b)
EC (us/cm)		23.80 ± 7.17 (a)	163.75 ± 35.65 (b)	70.42 ± 11.10 (a)
C/N		9.16 ± 0.49 (a)	13.10 ± 0.28 (b)	9.17 ± 1.00 (a)

Note: Different letters in the same line indicate a significant difference (*p* < 0.05).

**Table 2:** Alpha-diversity AVERAGE (standard deviation) of the soil microorganisms in the Poyang Lake Wetland, showing Observed Species, ACE, Chao1, Shannon, and Simpson indices.

Sample	Observed Species	ACE	Chao1	Shannon	Simpson	Pielou
S1	10,755 (687.89)	10,759.98 (687.44)	10,757.22 (687.65)	6.28 (0.17)	0.99 (0.002)	0.68 (0.016)
S2	13,403.33 (458.68)	13,413.72 (457.42)	13,407.63 (458.28)	6.23 (0.01)	0.99	0.64 (0.002)
S3	13,279 (83.48)	13,284.83 (83.59)	13,281.72 (83.05)	5.99 (0.02)	0.98 (0.001)	0.63 (0.002)

## Data Availability

The sequencing data set for this study was uploaded to the National Microbial Data Center of China (https://www.nmdc.cn/, accessed on 4 April 2023), and the BioProject number is NMDC20147401.

## Supplementary Materials

The following supporting Information can be downloaded at: https://www.mdpi.com/article/10.3390/microorganisms12122569/s1, Table S1. Basic information about metagenomic next-generation sequencing of soil samples from Poyang Lake Wetland; Figure S1. Correlation heatmap among various soil physicochemical properties in Poyang Lake wetland. The correlation range between physical and chemical properties is from −1 to 1, with blue representing positive values and red representing negative values. A p-value greater than 0.5 between pairwise properties is significant. The size of the circle represents the strength of the correlation; Figure S2. Histogram of soil microbial classification and their relative abundance in soil samples with different water content from Poyang Lake wetland. (a) Kingdom level (b) Class level (c) Order level (d) Family level; Figure S3. The β diversity PCA diagram of soil microbial communities in different water content samples from the Poyang Lake wetland. Different colors represent three soil samples with different moisture contents. (a) Diversity of microbial composition at the phylum level. The interpretation rate of PC1 and PC2 is 91.36%. (b) Diversity of microbial composition at the class level. The interpretation rate of PC1 and PC2 is 95.56%.

## References

[1] Nybo S.E., Khan N.E., Woolston B.M., Curtis W.R. (2015). Metabolic engineering in chemolithoautotrophic hosts for the production of fuels and chemicals. Metab. Eng..

[2] De S.L., Cabezas A., Marzorati M., Friedrich M.W., Boon N., Verstraete W. (2010). Microbial community analysis of anodes from sediment microbial fuel cells powered by rhizodeposits of living rice plants. Appl. Environ. Microbiol..

[3] Wu H., Wang X., He X., Zhang S., Liang R., Shen J. (2017). Effects of root exudates on denitrifier gene abundance, community structure and activity in a micro-polluted constructed wetland. Sci. Total Environ..

[4] Li T., Hu H., Wang J., Li Z., Lv X. (2016). Progress in research methods of soil microbial structure and diversity in wetlands. Chin. J. Soil. Sci..

[5] Zhang K., Li M., Yan Z., Li M., Kang E., Yan L., Zhang X., Li Y., Wang J., Yang A. (2022). Changes in precipitation regime lead to acceleration of the N cycle and dramatic N_2_O emission. Sci. Total Environ..

[6] Wang C., Yu J., Zhang J., Zhu B., Zhao W., Wang Z., Yang T., Yu C. (2024). A review of factors affecting the soil microbial community structure in wetlands. Environ. Sci. Pollut. Res. Int..

[7] Taş N., de Jong A.E., Li Y., Trubl G., Xue Y., Dove N.C. (2021). Metagenomic tools in microbial ecology research. Curr. Opin. Biotechnol..

[8] Fukuda K., Ogawa M., Taniguchi H., Saito M. (2016). Molecular approaches to studying microbial communities: Targeting the 16S ribosomal RNA gene. J. UOEH.

[9] Chiu C.Y., Miller S.A. (2019). Clinical metagenomics. Nat. Rev. Genet..

[10] Han D., Li Z., Li R., Tan P., Zhang R., Li J. (2019). mNGS in clinical microbiology laboratories: On the road to maturity. Crit. Rev. Microbiol..

[11] Gougoulias C., Clark J.M., Shaw L.J. (2014). The role of soil microbes in the global carbon cycle: Tracking the below-ground microbial processing of plant-derived carbon for manipulating carbon dynamics in agricultural systems. J. Sci. Food Agric..

[12] Salimi S., Almuktar S.A., Scholz M. (2021). Impact of climate change on wetland ecosystems: A critical review of experimental wetlands. J. Environ. Manag..

[13] Kirwan M.L., Megonigal J.P. (2013). Tidal wetland stability in the face of human impacts and sea-level rise. Nature.

[14] Bridgham S.D., Cadillo-Quiroz H., Keller J.K., Zhuang Q. (2013). Methane emissions from wetlands: Biogeochemical, microbial, and modeling perspectives from local to global scales. Glob. Chang. Biol..

[15] Jiang Y.M., Zhang C., Huang X.L., Ni C.Y., Wang J.F., Song P.F., Zhang Z.B. (2016). Effect of heavy metals in the sediment of Poyang Lake estuary on microbial communities structure base on Mi-seq sequencing. China Environ. Sci..

[16] Kou W., Zhang J., Lu X., Ma Y., Mou X., Wu L. (2016). Identification of bacterial communities in sediments of Poyang Lake, the largest freshwater lake in China. Springerplus.

[17] Yuan W., Chen L., Chen H., Deng S., Ji H., Liang F. (2023). Assessing habitat quality at Poyang Lake based on InVEST and Geodetector modeling. Ecol. Evol..

[18] Ren Q., Yuan J., Wang J., Liu X., Ma S., Zhou L., Miao L., Zhang J. (2022). Water level has higher influence on soil organic carbon and microbial community in Poyang Lake Wetland than vegetation type. Microorganisms.

[19] You Q., Yang W., Jian M., Hu Q. (2021). A comparison of metric scoring and health status classification methods to evaluate benthic macroinvertebrate-based index of biotic integrity performance in Poyang Lake wetland. Sci. Total Environ..

[20] Dai X., Yu Z., Yang G., Wan R. (2020). Role of flooding patterns in the biomass production of vegetation in a typical herbaceous wetland, Poyang Lake Wetland, China. Front. Plant Sci..

[21] Zhao M., Ma Y.T., He S.Y., Mou X., Wu L. (2020). Dynamics of bacterioplankton community structure in response to seasonal hydrological disturbances in Poyang Lake, the largest wetland in China. FEMS Microbiol. Ecol..

[22] Li B., Yang G., Wan R., Lai X., Wagner P.D. (2022). Impacts of hydrological alteration on ecosystem services changes of a large river-connected lake (Poyang Lake), China. J. Environ. Manag..

[23] Yu Y.W., Jorge G.M., Lin L.S., Min Z., Zhao S.W., Huan Z., Jun X. (2019). Drivers and changes of the Poyang Lake wetland ecosystem. Wetlands.

[24] Xu M., Li X., Kuyper T.W., Xu M., Li X., Zhang J. (2021). High microbial diversity stabilizes the responses of soil organic carbon decomposition to warming in the subsoil on the Tibetan Plateau. Glob. Chang. Biol..

[25] Moomaw W.R., Chmura G.L., Davies G.T., Finlayson C.M., Middleton B.A., Natali S.M., Perry J.E., Roulet N., Sutton-Grier A.E. (2018). Wetlands in a changing climate: Science, Policy and Management. Wetlands.

[26] Zhang N., Liu W., Yang H., Yu X., Gutknecht J.L., Zhang Z., Wan S., Ma K. (2013). Soil microbial responses to warming and increased precipitation and their implications for ecosystem C cycling. Oecologia.

[27] Li Y., Xiong L., Zeng K., Wei Y., Li H., Ji X. (2023). Microbial-driven carbon fixation in natural wetland. J. Basic Microbiol..

[28] Lennon J.T., Nguyễn-Thùy D., Phạm T.M., Drobniak A., Tạ P.H., Phạm N.D., Streil T., Webster K.D., Schimmelmann A. (2017). Microbial contributions to subterranean methane sinks. Geobiology.

[29] Chamberlain S.D., Anthony T.L., Silver W.L., Eichelmann E., Hemes K.S., Oikawa P.Y., Sturtevant C., Szutu D.J., Verfaillie J.G., Baldocchi D.D. (2018). Soil properties and sediment accretion modulate methane fluxes from restored wetlands. Glob. Chang. Biol..

[30] Qu Y., Zhao Y., Yao X., Wang J., Liu Z., Hong Y., Zheng P., Wang L., Hu B. (2023). Salinity causes differences in stratigraphic methane sources and sinks. Environ. Sci. Ecotechnol..

[31] He S., Malfatti S.A., McFarland J.W., Anderson F.E., Pati A., Huntemann M., Tremblay J., Glavina D.R.T., Waldrop M.P., Windham-Myers L. (2015). Patterns in wetland microbial community composition and functional gene repertoire associated with methane emissions. MBio.

[32] Kuypers M.M.M., Marchant H.K., Kartal B. (2018). The microbial nitrogen-cycling network. Nat. Rev. Microbiol..

[33] Ramond J.B., Jordaan K., Díez B., Heinzelmann S.M., Cowan D.A. (2022). Microbial biogeochemical cycling of nitrogen in arid ecosystems. Microbiol. Mol. Biol. Rev..

[34] Ortiz M., Bosch J., Coclet C., Johnson J., Lebre P., Salawu-Rotimi A., Vikram S., Makhalanyane T., Cowan D. (2020). Microbial nitrogen cycling in antarctic soils. Microorganisms.

[35] Zhang B.Y., Yu K. (2020). Application of microbial gene databases in the annotation of nitrogen cycle functional genes. Microbiol. China.

[36] Ma X.Y., Jiang L., Song Y.Y., Sun L., Song C.C., Hou A.X., Gao J.L., Du Y. (2021). Effects of temperature and moisture changes on functional gene abundance of soil nitrogen cycle in permafrost peatland. Acta Ecol. Sin..

[37] Davies J., Davies D. (2010). Origins and evolution of antibiotic resistance. Microbiol. Mol. Biol. Rev..

[38] Zhuang M., Achmon Y., Cao Y., Liang X., Chen L., Wang H., Siame B.A., Leung K.Y. (2021). Distribution of antibiotic resistance genes in the environment. Environ. Pollut..

[39] Zhou J., Chen Y., Qu J.H., Wang Y.K., Mai W.N., Wan D.J., Lu X.Y. (2022). Responses of microbial community and antibiotic resistance genes to co-existence of chloramphenicol and salinity. Appl. Microbiol. Biotechnol..

[40] Erwin K.L. (2009). Wetlands and global climate change: The role of wetland restoration in a changing world. Wetl. Ecol. Manag..

[41] Chen M., Wei X., Huang H., Lü T. (2011). Poyang Lake basin: A successful, large-scale integrated basin management model for developing countries. Water Sci. Technol..

[42] Xiang Y., Wang Y., Zhang C., Shen H., Wang D. (2018). Water level fluctuations influence microbial communities and mercury methylation in soils in the Three Gorges Reservoir, China. J. Environ. Sci..

[43] Anthony T.L., Silver W.L. (2020). Mineralogical associations with soil carbon in managed wetland soils. Glob. Chang. Biol..

[44] Adomako M.O., Xue W., Tang M., Du D.L., Yu F.H. (2020). Synergistic effects of soil microbes on solidago canadensis depend on water and nutrient availability. Microb. Ecol..

[45] Li W., Feng D., Yang G., Deng Z., Rui J., Chen H. (2019). Soil water content and pH drive archaeal distribution patterns in sediment and soils of water-level-fluctuating zones in the East Dongting Lake wetland, China. Environ. Sci. Pollut. Res. Int..

[46] Xiao Y., Huang Z.G., Xiao H.X., Li Y.F., Peng W.X. (2021). Changes of soil microbial biomass carbon, nitrogen, and enzyme activities in East Dongting Lake wetlands at different water levels. Ying Yong Sheng Tai Xue Bao.

[47] Jansson J.K., Hofmockel K.S. (2020). Soil microbiomes and climate change. Nat. Rev. Microbiol..

[48] Hutchins D.A., Jansson J.K., Remais J.V., Rich V.I., Singh B.K., Trivedi P. (2019). Climate change microbiology—Problems and perspectives. Nat. Rev. Microbiol..

[49] Zhang H., Zheng S., Ding J., Wang O., Liu F. (2017). Spatial variation in bacterial community in natural wetland-river-sea ecosystems. J. Basic Microbiol..

[50] Cheung M.K., Wong C.K., Chu K.H., Kwan H.S. (2018). Community structure, dynamics and interactions of bacteria, archaea and fungi in subtropical coastal wetland sediments. Sci. Rep..

[51] Zhou Z., Meng H., Liu Y., Gu J.D., Li M. (2017). Stratified bacterial and archaeal community in mangrove and intertidal wetland mudflats revealed by high throughput 16S rRNA gene sequencing. Front. Microbiol..

[52] Guo J., Wang X., Cao X., Qi W., Peng J., Liu H., Qu J. (2023). The influence of wet-to-dry season shifts on the microbial community stability and nitrogen cycle in the Poyang Lake sediment. Sci. Total Environ..

[53] Ma Y., Li J., Wu J., Kong Z., Feinstein L.M., Ding X., Ge G., Wu L. (2018). Bacterial and fungal community composition and functional activity associated with lake wetland water level gradients. Sci. Rep..

[54] Liu Y.J., Liu X., Mou X.Z., Wu L. (2019). Research status of microorganisms in a large, shallow lake Poyang Lake wetland. Microbiol. China.

[55] Sims A., Zhang Y., Gajaraj S., Brown P.B., Hu Z. (2013). Toward the development of microbial indicators for wetland assessment. Water Res..

[56] Mellado M., Vera J. (2021). Microorganisms that participate in biochemical cycles in wetlands. Can. J. Microbiol..

[57] Sánchez O. (2017). Constructed wetlands revisited: Microbial diversity in the -omics era. Microb. Ecol..

[58] Bar-On Y.M., Milo R. (2019). The global mass and average rate of rubisco. Proc. Natl. Acad. Sci. USA.

[59] Uchino Y., Yokota A. (2003). “Green-like” and “red-like” RubisCO *cbbL* genes in *Rhodobacter azotoformans*. Mol. Biol. Evol..

[60] Cai C., Leu A.O., Xie G.J., Guo J., Feng Y., Zhao J.X., Tyson G.W., Yuan Z., Hu S. (2018). A methanotrophic archaeon couples anaerobic oxidation of methane to Fe(III) reduction. ISME J..

[61] Leu A.O., Cai C., McIlroy S.J., Southam G., Orphan V.J., Yuan Z., Hu S., Tyson G.W. (2020). Anaerobic methane oxidation coupled to manganese reduction by members of the *Methanoperedenaceae*. ISME J..

[62] Venturini A.M., Dias N.M.S., Gontijo J.B., Yoshiura C.A., Paula F.S., Meyer K.M., Nakamura F.M., França A.G.D., Borges C.D., Barlow J. (2022). Increased soil moisture intensifies the impacts of forest-to-pasture conversion on methane emissions and methane-cycling communities in the Eastern Amazon. Environ. Res..

[63] Galloway J.N., Townsend A.R., Erisman J.W., Bekunda M., Cai Z., Freney J.R., Martinelli L.A., Seitzinger S.P., Sutton M.A. (2008). Transformation of the nitrogen cycle: Recent trends, questions, and potential solutions. Science.

[64] Wang B., Zhao J., Guo Z., Ma J., Xu H., Jia Z. (2015). Differential contributions of ammonia oxidizers and nitrite oxidizers to nitrification in four paddy soils. ISME J..

[65] Larsson D.G.J., Flach C.F. (2022). Antibiotic resistance in the environment. Nat. Rev. Microbiol..

[66] Su Z., Wen D. (2022). Characterization of antibiotic resistance across Earth’s microbial genomes. Sci. Total Environ..

