# Supplementary material for: Metagenomic Analysis Revealing the Impact of Water Contents on the Composition of Soil Microbial Communities and the Distribution of Major Ecological Functional Genes in Poyang Lake Wetland Soil
Source: Microorganisms. 2024 Dec 13;12(12):2569. doi: 10.3390/microorganisms12122569 (PMC11677957; doi:10.3390/microorganisms12122569)
Supplement: Supplementary file 1 [file microorganisms-12-02569-s001.zip › microorganisms-3363322-supplementary.pdf]

Metagenomic Analysis Revealing the Impact of Water Contents on the Composition of Soil Microbial Communities and the Distribution of Major Ecological Functional Genes in Poyang Lake Wetland Soil

Yuxin Long, Xiaomei Zhang, Xuan Peng, Huilin Yang, Haiyan Ni, Long Zou and Zhong'er Long *

Nanchang Key Laboratory of Microbial Resources Exploitation & Utilization from Poyang Lake Wetland, College of Life Sciences, Jiangxi Normal University, Nanchang 330022, China; longyuxin04@163.com (Y.L.); yanghl@jxnu.edu.cn (H.Y.); nihaiyan16@163.com (H.N.)

* Correspondence: longzhonger@163.com

Table S1. Basic information about metagenomic next-generation sequencing of soil samples from Poyang Lake Wetland.

Scheme 50.	Raw reads	Clean reads	N50	GC content (%)
S1	66,361,603	66,359,298	653	56.37
S2	65,391,661	65,387,372	860.6	63.84
S3	68,294,339	68,293,247	770	58.3

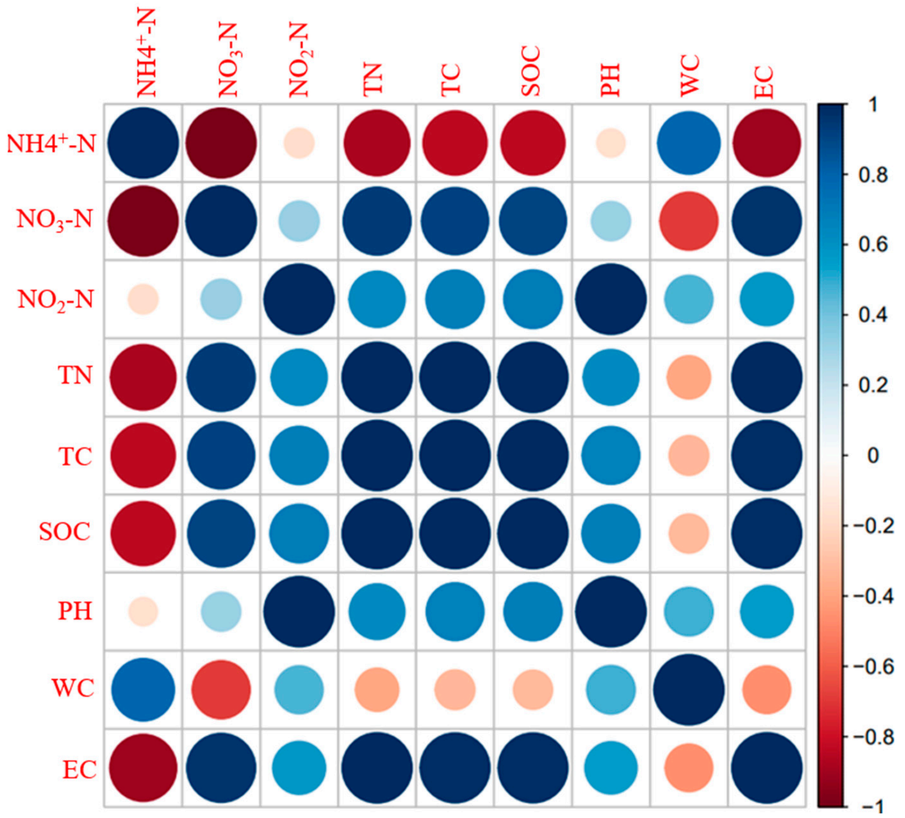

Figure S1. Correlation heatmap among various soil physicochemical properties in Poyang Lake wetland. The correlation range between physical and chemical properties is from -1 to 1, with blue representing positive values and red representing negative values. A p-value greater than 0.5 between pairwise properties is significant. The size of the circle represents the strength of the correlation.

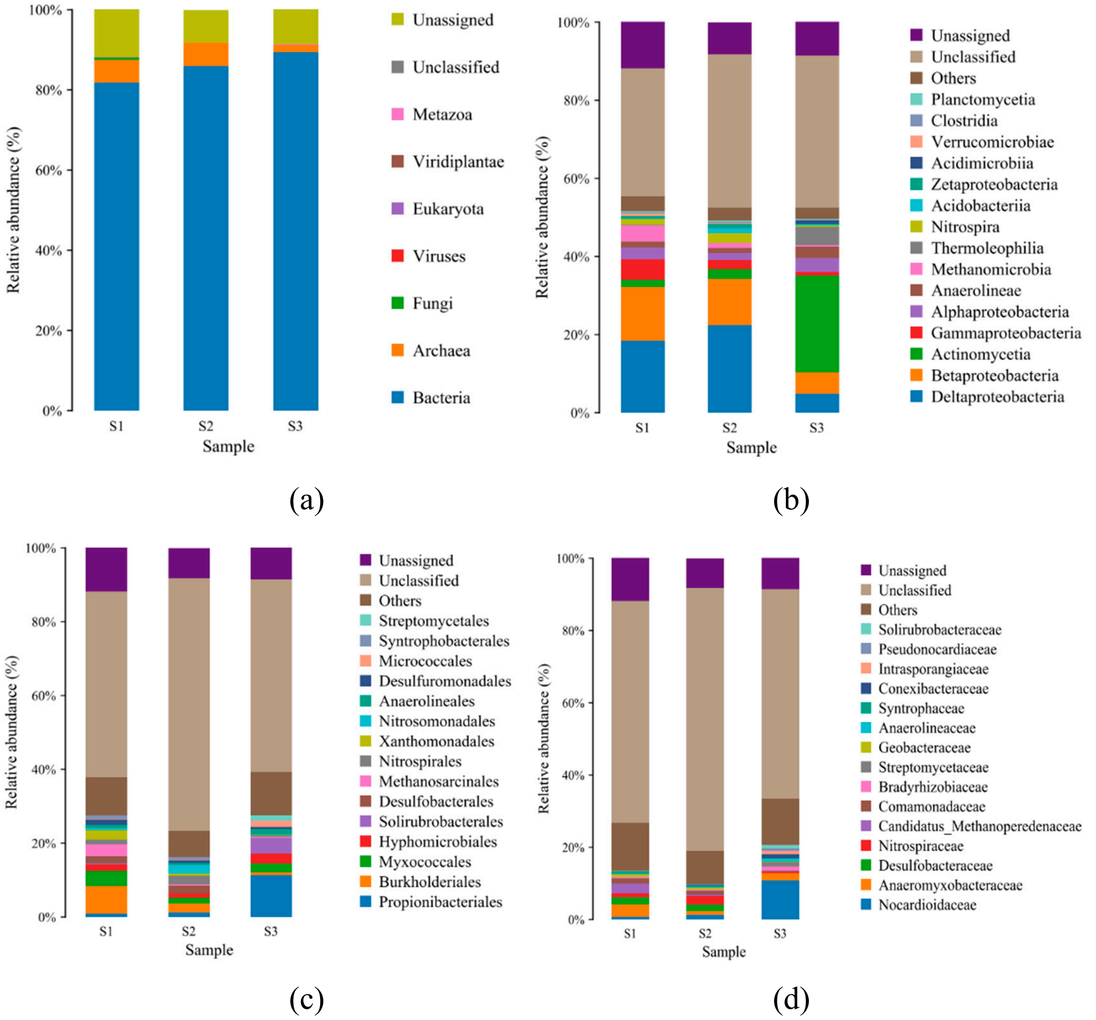

Figure S2. Histogram of soil microbial classification and their relative abundance in soil samples with different water content from Poyang Lake wetland. (a) Kingdom level (b) Class level (c) Order level (d) Family level.

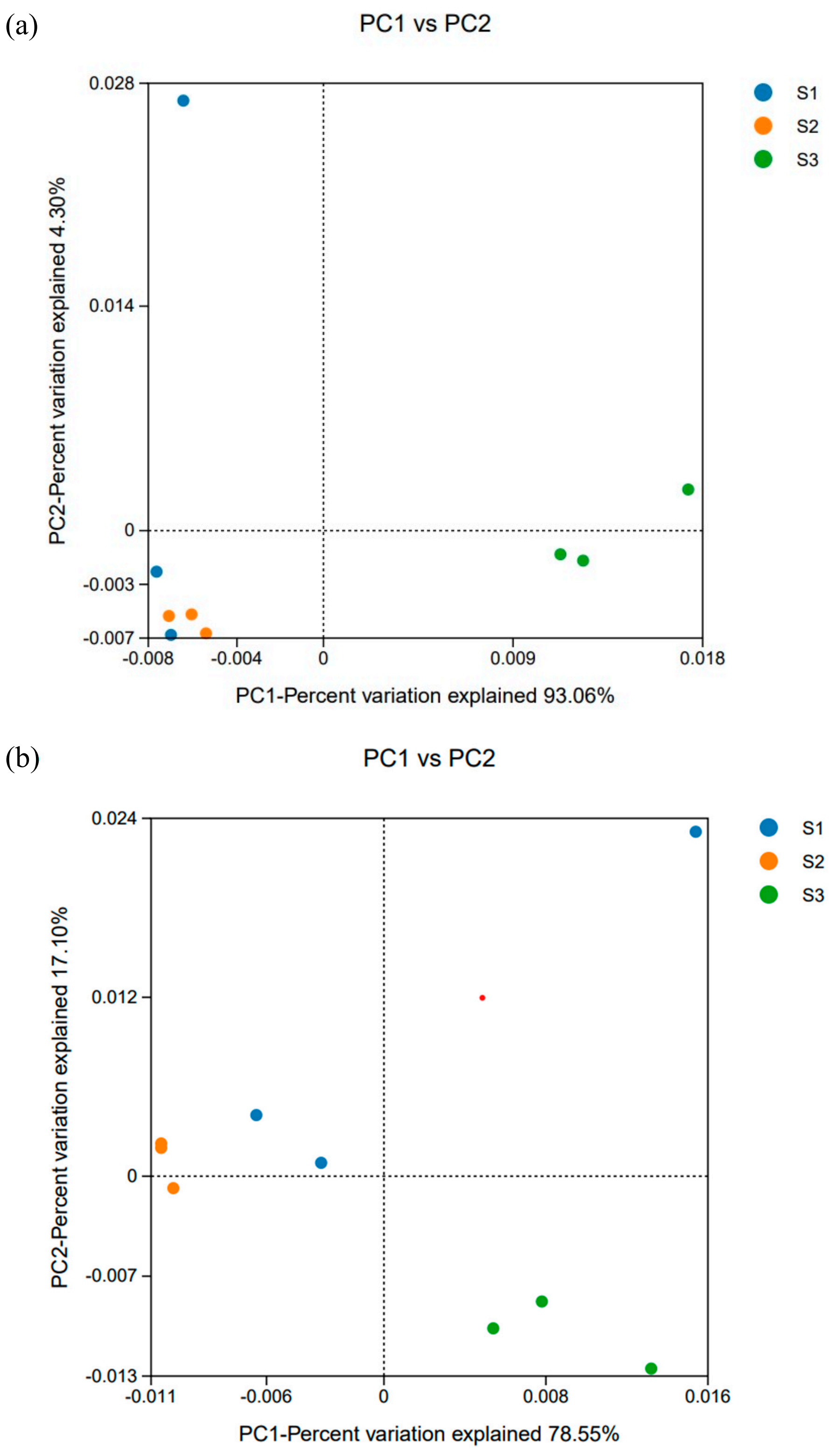

Figure S3. The β diversity PCA diagram of soil microbial communities in different water content samples from the Poyang Lake wetland. Different colors represent three soil samples with different moisture contents. (a) Diversity of microbial composition at the phylum level. The interpretation rate of PC1 and PC2 is 91.36%. (b) Diversity of microbial composition at the class level. The interpretation rate of PC1 and PC2 is 95.56%.
